# Sex differences in out-of-hospital cardiac arrest

**DOI:** 10.18632/aging.102980

**Published:** 2020-04-03

**Authors:** Iris Oving, Marieke T. Blom, Hanno L. Tan

**Affiliations:** 1 Department of Clinical and Experimental Cardiology, Amsterdam UMC, University of Amsterdam, Amsterdam, The Netherlands; 2 Netherlands Heart Institute, Utrecht, The Netherlands

**Keywords:** ESCAPE-NET, out-of-hospital cardiac arrest, sex, incidence, cardiopulmonary resuscitation, survival

An out-of-hospital cardiac arrest (OHCA) occurs when the heart stops maintaining its function to pump blood through the body in an out-of-hospital setting, typically due to cardiac arrhythmia. Our recent analysis of sex differences in OHCA incidence, treatment and survival in a large cohort of OHCA patients in the Netherlands demonstrated several important findings across various age groups [1]. First, although OHCA occurs almost equally as often in women as in men (women: 47.3%, men: 52.7%), women have significantly smaller chances of receiving resuscitation treatment by Emergency Medical Services (EMS) than men (women: 14.8%, men: 34.6%). This difference occurred in the age groups ≥50 years, and increased with age. Second, among EMS-treated patients, women with OHCA were less likely than men to receive bystander cardiopulmonary resuscitation (CPR) (women: 67.9%, men: 72.7%), even when OHCA was witnessed (women: 69.2%, men: 73.9%), consistent with a prior study [2]. Third, if resuscitation by EMS was attempted, women benefit less than men. This is reflected in lower overall survival to hospital discharge across all age groups (women: 12.5%, men: 20.1%), survival from OHCA to hospital admission (women: 33.6%, men: 36.6%), and survival from hospital admission to hospital discharge (women: 37.2%, men: 54.8%). However, when limited to patients with a shockable initial rhythm (SIR), overall survival to hospital discharge did not differ between sexes.

A SIR (ventricular tachycardia or ventricular fibrillation) is a very fast heart rhythm resulting in no effective pump function (i.e. blood can no longer circulate around the body). If SIR is not shocked back to normal with a defibrillator, it dissolves within minutes into asystole ('flat line') [3]. At this stage, defibrillation is no longer effective and death ensues within minutes. The swiftness of the pre-hospital resuscitation response therefore importantly determines the likelihood of presence of SIR upon connection of a defibrillator. Accordingly, presence of SIR is strongly influenced by OHCA location and presence/absence of a witness or bystander CPR. We observed that lower proportions of SIR occurred in women than in men, even after adjustment for patient and resuscitation characteristics and despite similar delays from EMS call to recognition of OHCA by the dispatcher. This lower rate of SIR in women contributes strongly to the lower survival rate and may be caused by longer delay from OHCA onset to recognition by bystanders (and call to EMS dispatcher) or by a more rapid transition from SIR into asystole due to biologic factors, or both.

Recognition by bystanders may be longer in women with OHCA because signs of impending OHCA may be different. For instance, the symptoms of myocardial infarction (MI), a common cause of OHCA, may not be recognised so quickly in women, as women may have nonspecific symptoms such as fatigue, fainting, vomiting and neck or jaw pain [4]. In contrast, men are more likely to report typical complaints such as chest pain. Also, biological differences between sexes in cardiac function are increasingly being recognized. For instance, electrophysiological differences may partly explain the lower rate of SIR in women. In particular, myocardial action potential duration shortens during MI and this process delays the onset of irreversible ischemic damage (which is signalled by loss of a shockable initial rhythm and onset of asystole) [5]. The shortening of action potential duration is attenuated in women because women have lower expression of repolarizing potassium channels than men [6].

How do the differences between men and women evolve with increasing age? We observed differences between age groups for OHCA incidence, EMS treatment and survival after OHCA. While increasing age is associated with rising OHCA incidence in both sexes, it brings about an increasing disadvantage suffered by women in receiving EMS resuscitation attempts. This is especially relevant, since the majority of OHCA occurs in patients aged >65 years. The finding that older women are less likely to receive an EMS resuscitation attempt may be explained by the higher life expectancy of women, leading to a higher likelihood of being widowed and/or living alone. Persons living alone are more likely to die unwitnessed, because they have much smaller chances of timely resuscitation treatment. With regards to survival chances, we observed that, among patients with SIR, survival chances were higher in women aged <50 years than in same-aged men. Studies suggested that women of reproductive age had better survival rates than women of higher ages or men, possibly due to elevated levels of female sex hormones [7]. Another study showed that the probability of survival decreased in male OHCA patients only after they reach an age of 65 years. Women, in contrast, have a constant decrease in survival with increasing age [8]. Our results seem to be in line with this finding.

Our findings may have important implications. In particular, the observation that women have a lower chance on a resuscitation attempt might indicate that initial recognition of OHCA in women is lagging behind. It would suggest that people may be less aware that OHCA can occur as often in women as in men, and that women themselves may not recognise the urgency of their symptoms and seek timely help. This provides the incentive for education campaigns and reorganization of health care, e.g. development of systems for quicker access to resuscitation care for elderly women living alone such as remote monitoring systems or provision of more automated external defibrillators at residences of single elderly women.
